# Comprehensive Transcriptome Analysis Reveals Accelerated Genic Evolution in a Tibet Fish, *Gymnodiptychus pachycheilus*

**DOI:** 10.1093/gbe/evu279

**Published:** 2014-12-26

**Authors:** Liandong Yang, Ying Wang, Zhaolei Zhang, Shunping He

**Affiliations:** ^1^The Key Laboratory of Aquatic Biodiversity and Conservation of Chinese Academy of Sciences, Institute of Hydrobiology, Chinese Academy of Sciences, Wuhan, Hubei, People’s Republic of China; ^2^University of Chinese Academy of Sciences, Beijing, People’s Republic of China; ^3^Department of Molecular Genetics, University of Toronto, Ontario, Canada; ^4^Donnelly Centre for Cellular and Biomolecular Research, University of Toronto, Ontario, Canada

**Keywords:** Tibetan Plateau, adaptation, positive selection, schizothoracine fish, transcriptome

## Abstract

Elucidating the genetic mechanisms of organismal adaptation to the Tibetan Plateau at a genomic scale can provide insights into the process of adaptive evolution. Many highland species have been investigated and various candidate genes that may be responsible for highland adaptation have been identified. However, we know little about the genomic basis of adaptation to Tibet in fishes. Here, we performed transcriptome sequencing of a schizothoracine fish (*Gymnodiptychus pachycheilus*) and used it to identify potential genetic mechanisms of highland adaptation. We obtained totally 66,105 assembled unigenes, of which 7,232 were assigned as putative one-to-one orthologs in zebrafish. Comparative gene annotations from several species indicated that at least 350 genes lost and 41 gained since the divergence between *G. pachycheilus* and zebrafish. An analysis of 6,324 orthologs among zebrafish, fugu, medaka, and spotted gar identified consistent evidence for genome-wide accelerated evolution in *G. pachycheilus* and only the terminal branch of *G. pachycheilus* had an elevated Ka/Ks ratio than the ancestral branch. Many functional categories related to hypoxia and energy metabolism exhibited rapid evolution in *G. pachycheilus* relative to zebrafish. Genes showing signature of rapid evolution and positive selection in the *G. pachycheilus* lineage were also enriched in functions associated with energy metabolism and hypoxia. The first genomic resources for fish in the Tibetan Plateau and evolutionary analyses provided some novel insights into highland adaptation in fishes and served as a foundation for future studies aiming to identify candidate genes underlying the genetic bases of adaptation to Tibet in fishes.

## Introduction

Understanding how species adapt to extreme environments is a central goal in evolutionary biology (Smith and Eyre-Walker 2002). As the world’s highest and largest plateau, the Tibetan Plateau, with an average elevation of 4,500 m above sea level, imposes many inhospitable living conditions on most organisms, including cold temperatures, low oxygen concentrations, and strong ultraviolet radiation (Thompson et al. 2000; Bickler and Buck 2007). Nevertheless, several species have well adapted to these harsh living challenges. Indeed, recent genome-wide studies on multiple species have identified various adaptive processes that may be responsible for highland adaptation, including humans (Beall et al. 2010; Bigham et al. 2010; Simonson et al. 2010; Yi et al. 2010; Peng et al. 2011; Xu et al. 2011; Xing et al. 2013), yak (Qiu et al. 2012), Tibetan antelope (Ge et al. 2013), the ground tit (Cai et al. 2013; Qu et al. 2013), and Tibetan Mastiff (Gou et al. 2014; Li et al. 2014). Among these adaptive processes, it was well-known that genes showing signals of positive selection and expansion were significantly enriched in hypoxia-inducible factor (HIF) and energy metabolic pathways. For example, hypoxia-related genes (such as *EPAS1*, *EGLN1*, and *PPARA*) have experienced strongly positive selection and are significantly associated with the decreased hemoglobin concentration in Tibetans (Beall et al. 2010; Simonson et al. 2010; Yi et al. 2010). However, almost all previous genome-wide studies were performed on endothermic terrestrial vertebrates. We know little about the genomic bases of adaptation to highland in fishes. Therefore, it may provide some novel insights by investigating the genetic mechanisms of adaptation to the Tibetan Plateau in fishes.

The schizothoracine fishes (Teleostei: Cyprinidae), which are distributed throughout the Tibetan Plateau and its peripheral regions, are the largest and most diverse taxon of the Tibetan Plateau ichthyofauna (Cao et al. 1981; Chen and Cao 2000). These fishes are the only taxon within the most successful family Cyprinidae that have well adapted to the hostile environment of the Tibetan Plateau (He et al. 2004). The schizothoracine fishes dominate the plateau lakes and torrential mountain streams of the Tibetan Plateau (He and Chen 2006) and have evolved a number of unique traits to adapt to the hypoxia and cold environment (Wu Y and Wu C 1991). Therefore, they have been considered as excellent models to investigate high altitude adaptation of fishes. According to the degree of specialization of the scales, pharyngeal teeth, and barbels, the schizothoracine fishes are divided into three grades: Primitive, specialized, and highly specialized schizothoracine fishes and the orderly reductions of these morphological characteristics in these groups were closely associated with the drastic environmental changes caused by three stages of violent upheaval of the Tibetan Plateau (Cao et al. 1981). Thus, it was suggested that the three phases of uplift of the Tibetan Plateau have contributed to the speciation of the schizothoracine fishes. The species *Gymnodiptychus pachycheilus*, belonging to the specialized schizothoracine fishes, distributes only in the headwater area in the northeast of the Tibetan Plateau with elevations of 2,750–3,750 m (He et al. 2004) and is the most dominant group of the ichthyofauna of the Yellow River (Wu Y and Wu C 1991). However, human activities, including overexploitation and habitat destruction, have affected this species considerably, which makes *G. pachycheilus* listed as an endangered species in the “China Species Red List” (Wang and Xie 2004). Thus, characterization and evolutionary analyses of its transcriptome resources can not only provide information of highland adaptation of fishes but also help protect its population.

The recent rapid advances in sequencing technologies have offered the opportunity to generate transcriptomes in almost any species of interest. When genome sequence is not available, transcriptome sequencing is a rapid and effective approach to obtain massive protein-coding genes and molecular makers. In this study, we generated the first transcriptome of a schizothoracine fish (*G. pachycheilus*) endemic to the Tibetan Plateau using high-throughput sequencing technology. We then characterized the transcriptome comprehensively and performed evolutionary analyses together with other previously available fish genomes to investigate the potential mechanisms of highland adaptation of fishes.

## Materials and Methods

### Fish Sampling, RNA Extraction, and Sequencing

All animal experiments were performed in accordance with the ethics committee of Institute of Hydrobiology, Chinese Academy of Sciences. One wild schizothoracine fish (*G. pachycheilus*) was sampled from Gansu Fisheries and Science Research Institute, Lanzhou, Gansu, China. To obtain as many expressed genes as possible, five different types of organs (heart, brain, liver, kidney, and spleen) were sampled and stored in RNAlater (QIAGEN) immediately. Total RNA was isolated using the SV Total RNA Isolation System (Promega) according to the manufacturer’s protocol and the quality of RNA was measured using electrophoresis and the BioPhotometer plus 6132 (Eppendorf, Germany). Poly (A) mRNA was purified using Oligo (dT) magnetic beads and interrupted into short fragments. Subsequently, the first-strand cDNA was synthesized using random hexamer primer and then second-strand cDNA was generated. Finally, the paired-end cDNA library was prepared according to the Illumina’s protocols and sequenced (101 bp read length) on Illumina HiSeq 2000 platform. The sequencing data have been deposited into the National Center for Biotechnology Information (NCBI) Sequence Read Archive database (Accession No. SRR1583887)

### De Novo Assembly

The raw reads were first preprocessed and filtered by removing reads with sequencing adaptors, reads with unknown nucleotides and low quality (quality scores < 20). All subsequent analyses were based on these filtered reads. Next, transcriptome de novo assembly was performed using Trinity software (Grabherr et al. 2011) with default parameters. Only contigs longer than 200 bp were kept for further analysis. Then, CD-HIT-EST program (Li and Godzik 2006) was used to further remove the redundancy in the final assembly.

### Gene Annotation

To annotate the assembled unigenes, we first downloaded the protein data sets of zebrafish (*Danio rerio*) from the Ensembl database (release-75) (Flicek et al. 2013) and then using BLASTX (Altschul et al. 1997) searches to map the unigenes to these proteins with an *E* value cutoff of 1 × 10^−^^10^. In order to identify genes that may be lost (or missing) in the zebrafish genome, unigenes without hits against zebrafish proteins were used to search against protein data sets from other model fishes *Astyanax mexicanus*, *Gadus morhua*, *Gasterosteus aculeatus*, *Oreochromis niloticus*, *Oryzias latipes*, *Takifugu rubripes*, *Tetraodon nigroviridis*, and *Xiphophorus maculatus* from the Ensembl database. Then, those unigenes with hits in other model fishes were further searched against the zebrafish genome with BLASTN and BLAT (Kent 2002) to confirm that these putative genes were lost in the zebrafish genome. Putative functions for assembled unigenes were assigned by BLAST2GO suit (Gotz et al. 2008) using BLASTX against the nonredundant (NR) databases with a conservative *E* value cutoff of 1 × 10^−^^5^. We then extracted the Open reading frames (ORFs) using getorf tool implemented in EMBOSS (Rice et al. 2000) and predicted the protein-coding potential for the assembled unigenes using CPAT (Wang et al. 2013), with Zebrafish (Zv9/danRer7) as the assembly database and 0.38 as the coding probability cutoff.

### Identification of Orthologs

Putative orthologs between *G. pachycheilus* and zebrafish were determined using the reciprocal BLAST best-hit method with an *E* value cutoff of 1 × 10^−^^10^. Then one-to-one orthologs between zebrafish, fugu, medaka, and spotted gar (*Lepisosteus oculatus*) were obtained from Ensembl using Biomart (Durinck et al. 2005). When genes had multiple transcripts, the longest one was used. Each orthologous gene set was aligned using PRANK (Loytynoja and Goldman 2005) with the parameter “-codon” and trimmed using GBlocks (Castresana 2000) with the parameter “-t = c.” We further deleted all gaps and “N” from the alignments to lower the effect of ambiguous bases on the inference of positive selection. After the deletion process, the trimmed alignments shorter than 150 bp (50 codons) were discarded for subsequent analyses.

### Substitution Rate Estimation and Selection Analyses

To estimate lineage-specific evolutionary rates for each branch of the five species, the Codeml program in the PAML package (Yang 2007) with the free-ratio model (model = 1) was run on each ortholog, a concatenation of all alignments of the orthologs, and 1,000 concatenated alignments constructed from ten randomly chosen orthologs. Parameters, including d*N*, d*S*, d*N*/d*S*, N*d*N*, and S*d*S* values, were obtained for each branch and genes were discarded if N*d*N* or S*d*S* < 1, or d*S* >2, according to previous study (Goodman et al. 2009).

We used the branch model to identify fast evolving genes (FEGs) with the null model assuming that all branches have been evolving at the same rate and the alternative model allowing foreground branch to evolve under a different rate. The likelihood ratio test (LRT) with df = 1 was used to discriminate between alternative model for each ortholog in the gene set. Multiple testing was corrected by applying the false discovery rate (FDR) method implemented in R (Storey and Tibshirani 2003). We considered the genes as evolving with a significantly faster rate in foreground branch if the FDR-adjusted *P* value less than 0.05 and a higher ω values in the foreground branch than the background branches.

To detect positive selection on a few codons along specific lineage, we used the optimized branch-site model (Zhang et al. 2005) following the author’s recommendation. A LRT was constructed to compare a model that allows sites to be under positive selection on the foreground branch with the null model in which sites may evolve neutrally and under purifying selection. The *P* values were computed based on the Chi-square statistic adjusted by the FDR method and genes with adjusted *P* value less than 0.05 were treated as candidates for positive selection.

Gene ontology (GO) functional enrichment analyses for both FEGs and positively selected genes (PSGs) were carried out by DAVID (Dennis et al. 2003; Huang da et al. 2009).

## Results

### Sequence Analysis and Assembly

A mixed sample of cDNAs obtained from five tissues, including heart, brain, liver, kidney, and spleen, was prepared and sequenced using the Illumina HiSeq 2000 platform, which produced 22,805,393 raw 101-bp paried-end reads. After removing adaptors and low-quality reads, we obtained 22,728,725 quality filtered reads pairs with a median read length of 100 bp. With these high quality reads, 132,794 reconstructed contigs were generated using Trinity, with a median length of 745 bp and an N50 of 2,322 bp. We further used CD-HIT-EST to produce an NR unigene data sets and obtained 66,105 unigenes ranging from 201 to 21,730 bp, with a median length of 710 bp and an N50 of 1,602 bp (supplementary table S1, Supplementary Material online). The length distribution of all unigenes is provided and nearly 60% of the unigenes are between 200 and 500 bp. (supplementary fig. S1, Supplementary Material online). A significantly positive relationship between the length of unigenes and number of reads covered was observed, with an average coverage depth of 263 reads (supplementary fig. S2, Supplementary Material online).

To assess the quality of our assembled unigenes, we downloaded all 13 mitochondrial protein-coding genes available for *G. pachycheilus* from NCBI database as reference sequences and compared our assembled unigenes with these reference genes using BLASTN with an *E* value cutoff of 1 × 10^−^^10^. All these protein-coding genes were found to be present in our assembled unigenes. The proportions of mismatching nucleotides relative to the reference sequences were calculated and only 0.74% mean nucleotide difference was observed. We further obtained the complete mitochondrial genome sequence (Wu et al. 2014) to evaluate the completeness and continuity of our assembled unigenes. We found a total of 15,429 nucleotide identities out of 15,514 (99.5%) total nucleotide length of unigene relative to whole mitochondrial sequences. In addition to above computing method, we further performed reverse transcription polymerase chain reaction to validate the quality of our assembled unigenes. We randomly picked ten unigenes with different expression levels (reads per kilobase per million mapped [RPKM] ranged from 51 to 889). Primers for these unigenes were designed and all these cDNAs can be successfully amplified (supplementary table S2 and fig. S3, Supplementary Material online). These results demonstrated reliable transcriptome assembly quality, which is the foundation for subsequently comparative genomic analysis.

### Functional Annotation

We used several complementary approaches to annotate the assembled unigenes. First, a BLASTX search against zebrafish proteins returned 28,586 (43.2%) *G. pachycheilus* unigenes with significant hits to zebrafish genes. This percentage of unigenes with BLAST hits is comparable with previous de novo transcriptome studies for nonmodel organisms (Guo et al. 2013), in which unigenes without significant hits may consist of orphan genes, noncoding RNAs, untranslated transcripts, or misassembled transcripts. Second, we used BLAST2GO with the GO annotation database to assign their putative functions and 24,131 unigenes have one or more GO terms (supplementary fig. S4, Supplementary Material online). Finally, Clusters of Orthologous Groups of protein databases were used to further annotate these unigenes and produced good results for 9,740 putative proteins (supplementary fig. S5, Supplementary Material online).

**Fig. 1.—:**
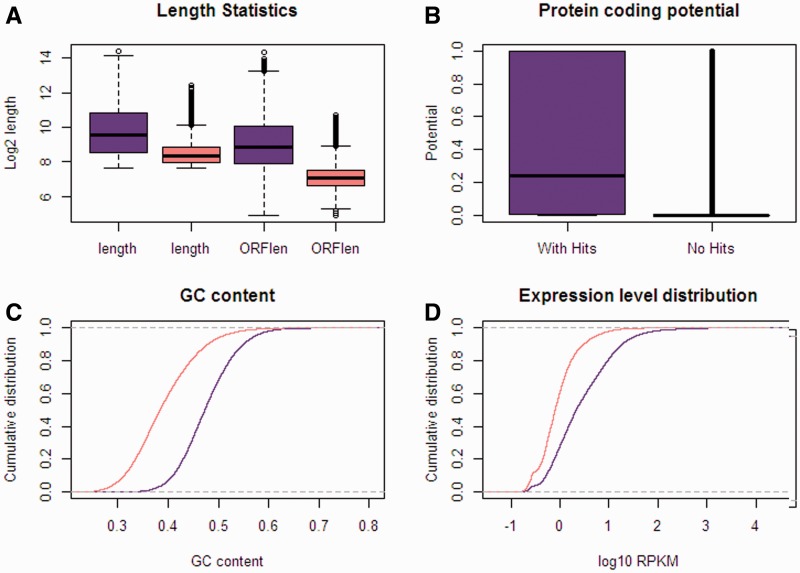
Comparison between the set of unigenes with and without BLAST-hits. (*A*) Overall length and longest ORF length statistics, (*B*) protein-coding potential determined by CPAT, (*C*) distribution of GC content, and (*D*) distribution of normalized expression level. Purple color, with BLAST hit; pink, without BLAST hit.

Homologs to known proteins of any species in the NR databases were identified for 31,733 unigenes which represent 48% of the total de novo reference transcriptome assembly. Overall, 11,058 unigenes had an *E* value of BLASTX results between 1 × 10^−^^5^ and 1 × 10^−^^50^ and 9,103 unigenes have an *E* value of 0. More than half (52.7%, *n* = 16,726) of the homologs to known proteins have identity between 80% and 100%. And 55.3% (*n* = 17,560) of the best hits were with zebrafish, which may reflect the close phylogenetic relationship between these two species, or reflect the wealthy genomic resources for zebrafish (supplementary fig. S6, Supplementary Material online). We next divided our assembly unigenes into two subsets (unigenes with and without protein homology in NR database, named as “with hits” and “no hits” set) and characterized their sequence and expression features in detail. Overall, the “with hits” set had significantly larger unigene length (median 758 vs. 318 bp) and longer ORFs (median 462 vs. 132 bp) than the “no hits” set (Wilcoxon rank sum test, *P* < 2.2 × 10^−^^5^) (fig. 1*A*). Analysis of the potential for protein coding with CPAT (Wang et al. 2013) revealed a significantly lower protein-coding potential in the group of unigenes without hits (Wilcoxon rank sum test, *P* < 2.2 × 10^−^^6^) (fig. 1*B*). The distributions of GC content and normalized expression level also show that, in general, unigenes with BLAST hits have higher values than those without hits (Wilcoxon rank sum test, *P* < 2.2 × 10^−^^6^) (fig. 1*C* and *D*). These characteristics between the “with hits” and “no hits” set of unigenes in *G. pachycheilus* were consistent with previous reports on nonmodel species without a reference genome (Ferreira et al. 2013; Schunter et al. 2014), which indicates that many novel unigenes may be nonprotein-coding sequences.

Additionally, we found that 1,065 unigenes, which did not have significant BLASTX hit against protein sequences from zebrafish, had significant hits against proteins from at least one of the other eight fish genomes obtained from Ensembl. After performing BLASTN and BLAT searches against zebrafish genome, 350 out of the 1,065 unigenes were confirmed to have no hits in the zebrafish genome (supplementary table S3, Supplementary Material online). Considering that these unigenes have orthologous genes in other teleost genomes, we thought that the orthologs of these unigenes are probably lost in the zebrafish genome instead of being gained in *G. pachycheilus*.

### Orphan Genes in *G. pachycheilus*

In the past few years, substantial progress has demonstrated that lineage-specific new genes can rapidly evolve indispensable biological roles and make a contribution to lineage-specific phenotype and adaptation (Chen et al. 2013). Thus, it is meaningful to identify putative novel protein-coding genes (orphan genes) in *G. pachycheilus*, which might have evolved specific functional roles and contributed to their adaptation to Tibetan plateau. To investigate this, we first predicted the protein-coding potential for each of the assembled unigenes in *G. pachycheilus* using the CPAT program. Out of the 66,105 assembled unigenes, 15,845 (24%) were predicted as protein-coding genes and these included 1,565 (10%) unigenes that had no identifiable zebrafish ortholog. To exclude any orthologs of these unigenes in other species, we further searched them against the NR databases and identified 744 with orthologs in any other species in NR databases. Among the remaining 821 unigenes, we set several cutoffs by calculating the median size (758 bp), the median protein-coding potential score (0.24), and the median expression level (RPKM = 2) of the 31,733 unigenes with identifiable orthologs against NR databases and detected 88 unigenes that were longer, had higher protein-coding potential, higher expression level than the median values of the unigenes with known protein-coding orthologs. Furthermore, as recommended by CPAT, 0.38 is the optimum cutoff to filter false protein-coding genes in fishes (Wang et al. 2013). Thus, we used this cutoff to further remove the candidate de novo protein-coding genes that have coding potential lower than 0.38. The remaining 88 unigenes were further searched against the zebrafish and other fish genome sequences and resulted in significant BLASTn hit for 47 unigenes. Finally, we identified 41 putative orphan genes specific to *G. pachycheilus*, which originated around 50 Ma after the split from zebrafish (Steinke et al. 2006) (supplementary table S4, Supplementary Material online).

### Accelerated Evolution on the Lineage Leading to Tibet Fish

To better understand the evolutionary dynamics of Tibet fish, we analyzed the putative single copy orthologs in *G. pachycheilus*, zebrafish, fugu, medaka, and spotted gar genomes. After alignment and trimming for quality control (see Materials and Methods), a total of 6,324 orthologs, ranging from 150 to 13,707 bp, were determined. Despite the lengths of the orthologs were shorter after trimming, their shapes of length distributions were generally similar (supplementary fig. S7, Supplementary Material online), which ensured subsequent evolutionary analyses.

**Fig. 2.—:**
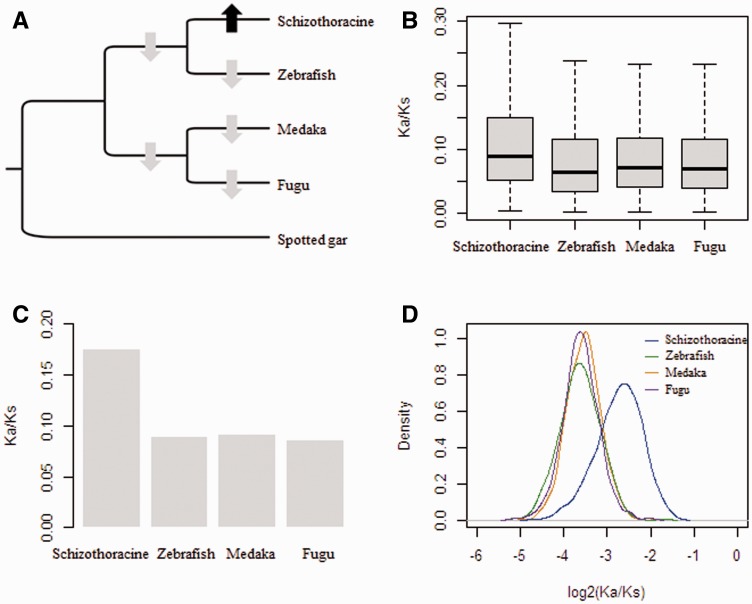
Phylogenetic tree used in this study (*A*) and branch specific Ka/Ks ratios obtained from different data sets (*B–D*). Gray and black arrows in (*A*) indicate decreased or increased terminal Ka/Ks ratios compared with the ancestral branch. The Ka/Ks ratios for terminal branches were estimated from each ortholog (*B*), concatenated all orthologs (*C*), and 1,000 concatenated alignments constructed from ten randomly chosen orthologs (*D*).

First, to compare the overall difference in selective constraints in different branch at the gene level, each orthologous gene was evaluated for substitution rates including Ka, Ks, and Ka/Ks, using the species tree (Near et al. 2012) (fig. 2*A*). The free-ratio model (M1 model) in PAML was used, which allows an independent Ka/Ks ratio for each branch (Yang 2007). Averaged across all 6,324 orthologous genes, the *G. pachycheilus* branch had a significantly higher ratio of nonsynonymous to synonymous substitutions than other fish branches (Wilcoxon rank sum test, *P* < 2.2 × 10^−^^16^), suggesting accelerated function evolution in the *G. pachycheilus* lineage (fig. 2*B*). Indeed, by examining the Ka/Ks ratio for each gene in the *G. pachycheilus* and zebrafish lineages, we found that 2,607 genes have higher Ka/Ks in *G. pachycheilus* whereas only 1,607 genes higher in zebrafish. We further calculated the Ka/Ks ratio for each branch for a concatenated alignment of all 6,324 orthologs and 1,000 concatenated alignments constructed from ten randomly chosen orthologs, and found that both data sets exhibited a significantly higher Ka/Ks ratio for the *G. pachycheilus* branch than other fish branches (Wilcoxon rank sum test, *P* < 2.2 × 10^−^^16^) (fig. 2*C* and *D*). Furthermore, comparison of Ka/Ks ratios between terminal and ancestral branches indicated that only the *G. pachycheilus* branch had an elevated Ka/Ks ratio than the ancestral branch (Wilcoxon rank sum test, *P* < 2.2 × 10^−^^16^) (fig. 2*A*), implying accelerated evolution only in *G. pachycheilus* after their split from zebrafish.

**Fig. 3.—:**
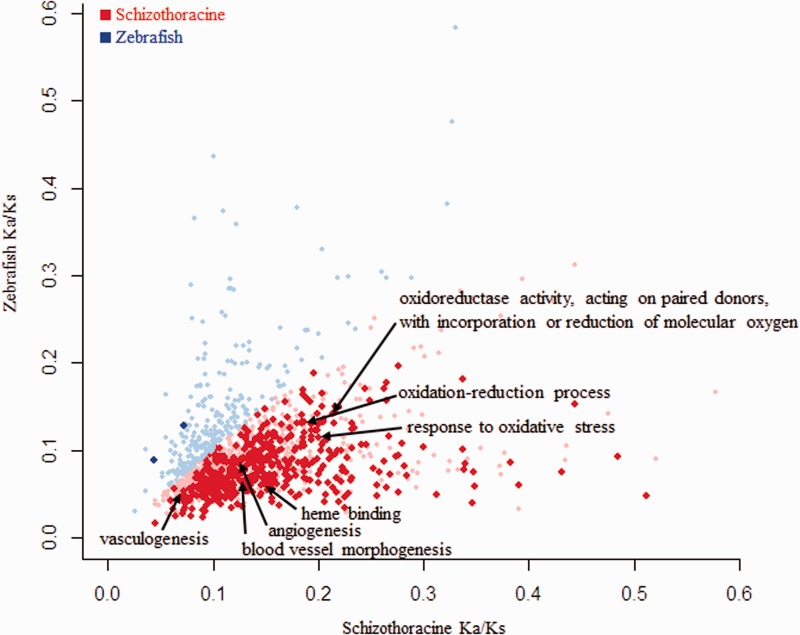
Scatter plot of mean Ka/Ks ratios for each GO category in *G. pachycheilus* and zebrafish. GO categories with significantly higher mean Ka/Ks ratios in *G. pachycheilus* (red) and zebrafish (blue) are highlighted. Light red and light blue points represent the GO categories with higher but not statistically significant mean Ka/Ks ratios in *G. pachycheilus* and zebrafish.

To identify the GO categories that undergone rapid or slow evolution in *G. pachycheilus* than zebrafish, we calculated the average Ka/Ks ratios for each GO category with at least ten orthologs in *G. pachycheilus* and zebrafish lineages, respectively. Among these GO categories, the number of GO category with average Ka/Ks ratios higher in *G. pachycheilus* lineage was significantly larger than the number of GO category with average Ka/Ks ratios higher in zebrafish lineage (1,108 vs. 258) and there was significantly larger number of GO categories with statistically significantly higher average Ka/Ks ratios in *G. pachycheilus* than in zebrafish lineage (480 vs. 2), confirming overall accelerated evolution in *G. pachycheilus*. Furthermore, many GO categories involved in energy metabolism, hypoxia response, and DNA repair showed significantly accelerated evolution in *G. pachycheilus* than zebrafish, such as “response to oxidative stress,” “blood vessel morphogenesis,” “glucose metabolic process,” “NAD binding,” and “positive regulation of DNA repair” (fig. 3 and supplementary table S5, Supplementary Material online).

### Fast Evolving and Positively Selected Genes

**Fig. 4.—:**
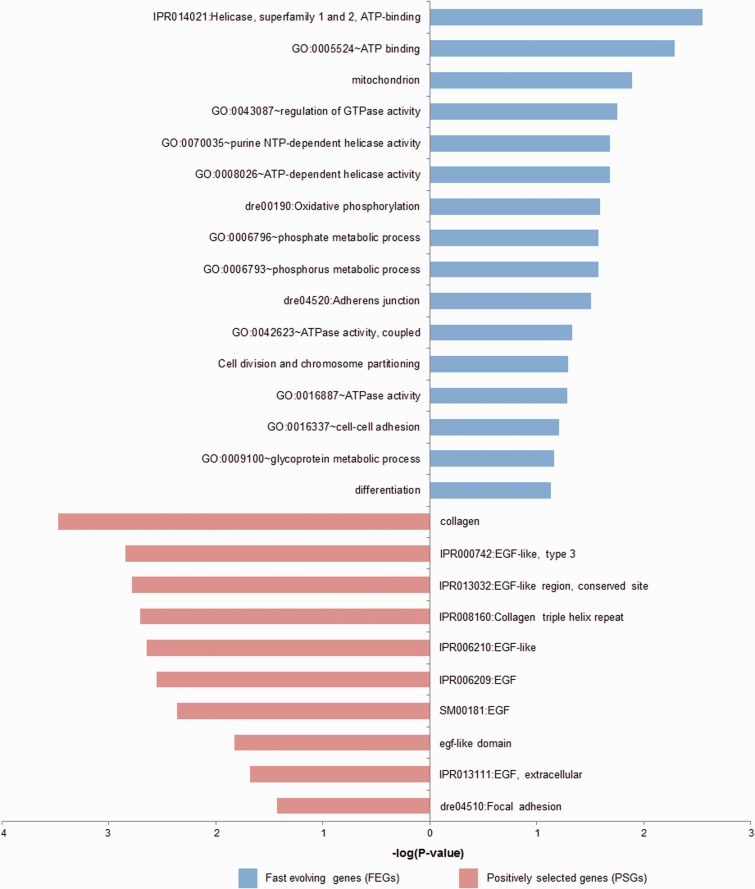
Functional enrichment analyses of FEGs and PSGs showing categories involved in energy metabolism and hypoxia response. Blue, FEGs; red, PSGs.

To detect genes that might evolve adaptively in specific lineage, two types of gene sets were compiled: 1) FEGs, which have experienced a significantly higher Ka/Ks ratio in specific lineage compared with other lineages, and 2) PSGs, which have been influenced by positive selection only on a few codons along particular lineage (see Materials and Methods). In total, we identified 883 FEGs in *G. pachycheilus* and 556 FEGs in zebrafish, and 123 PSGs in *G. pachycheilus* and 111 PSGs in zebrafish (supplementary tables S6 and S7, Supplementary Material online). Functional enrichment analysis showed that the FEGs identified in *G. pachycheilus* lineage were significantly enriched for genes involved in energy metabolism and oxidation-related functions, including “ATP binding,” “mitochondrion,” “regulation of GTPase activity,” and “Oxidative phosphorylation,” whereas FEGs detected in zebrafish were generally enriched in functions involved in structure components (fig. 4 and supplementary table S8, Supplementary Material online). Similarly, the PSGs identified in *G. pachycheilus* lineage rather than in zebrafish were also enriched for genes potentially related to hypoxia response, including epidermal growth factor (fig. 4 and supplementary table S9, Supplementary Material online). In addition, we found that the putative PSGs, whose *P* values were not corrected by FDR method, were also enriched for genes involved in adaptation to high-elevation environment, such as “vasculature development” (supplementary table S9, Supplementary Material online).

To identify genes that may directly contribute to the adaptation to high altitude, we combined two approaches to detect all the candidate genes according to their functional roles. First, we compared our candidate genes (PSGs) with an a priori list proposed by Zhang et al. (2014), which includes 1,351 putative hypoxia-related genes. Second, we made use of the functional annotated information for each PSG to identify the gene associated with hypoxia response reported in previous experimental studies. In total, we identified nine candidate PSGs in *G. pachycheilus* that may be involved in hypoxia response: BYSL, HSF1, YES1, ARRDC2, SSPN, SEMA4D, VWF, COMP, and LAMB (table 1).

**Table 1:** PSGs Involved in Hypoxia Response in *Gymnodiptychus pachycheilus*

Gene ID	Gene Name	Description	Adjusted *P* Value
ENSDARG00000001057	BYSL	Bystin-like	0.003
ENSDARG00000008818	HSF1	Heat shock transcription factor 1	0.03
ENSDARG00000005941	YES1	v-yes-1 Yamaguchi sarcoma viral oncogene homolog 1	0.04
ENSDARG00000020761	ARRDC2	Arrestin domain containing 2	0.02
ENSDARG00000041747	SSPN	Sarcospan (Kras oncogene-associated gene)	0.009
ENSDARG00000067801	SEMA4D	Sema domain, immunoglobulin domain (Ig), transmembrane domain (TM) and short cytoplasmic domain, (semaphorin) 4D	0.01
ENSDARG00000077231	VWF	von Willebrand factor	0.04
ENSDARG00000053865	COMP	Cartilage oligomeric matrix protein	0.03
ENSDARG00000059369	LAMB	Laminin, beta 3	0.001

## Discussion

Over the past few years, comparative genomics has been widely employed as a tool to understand the genetic bases of many fundamental evolutionary questions, including adaptation (Yi et al. 2010; Jones et al. 2012; Axelsson et al. 2013; Zhao et al. 2013), speciation (Ellegren et al. 2012; Poelstra et al. 2014; Soria-Carrasco et al. 2014), and genetic variation (Guo et al. 2012). When the genome sequencing data are not available, transcriptome sequencing is an effective and accessible approach to initiate comparative genomic analyses on nonmodel organisms, because they contain large number of protein-coding genes likely enriched for targets of natural selection. Here, using the next-generation sequencing technology, we have generated and annotated the first comprehensive transcriptome resources for a schizothoracine fish (*G. pachycheilus*), which is endemic to the Tibetan Plateau and shows many unique traits to adapt to highland environments (Wu Y and Wu C 1991; Su et al. 2014). We generated more than 7,000 pairwise orthologous genes between zebrafish and over 6,000 orthologous genes among other fish genomes, which are important bases for comparative genomic studies of adaptation in fishes. Therefore, the transcriptome resources produced by our study are useful to understand the genetic makeup of fishes in high altitude and provide a foundation for further studies to identify candidate genes underlying adaptation to the Tibetan Plateau of fishes.

Gene losses and gains are important adaptive processes that have a contribution to evolutionary innovations (Hahn et al. 2007; Ding et al. 2012). Thus, we first attempted to identify genes that present in *G. pachycheilus* but lost in zebrafish and genes gained specific in *G. pachycheilus* through comparison of orthologous genes between *G. pachycheilus* and other fish genomes. By setting a strict set of cutoffs, we revealed that as many as 350 genes have a potential to have been lost in zebrafish because they exist in both *G. pachycheilus* and other fish genomes. There are also alternative possibilities that these genes have evolved too fast to resemble their orthologs in other fishes, or that they are missed from the current zebrafish genome assembly. Among the genes that might have been lost in zebrafish, many have GO categories associated with binding, including RNA, protein, and nucleic acid binding, which is similar to potentially lost genes in three-spined sticklebacks (Guo et al. 2013). On the other hand, we identified at least 41 genes that are uniquely present in the *G. pachycheilus* transcriptome data set compared with other fishes. These genes are likely to have originated in the schizothoracine fish lineage around 50 Ma after split from the zebrafish (Steinke et al. 2006) and might have evolved novel functional roles that may be contributing to the adaptation to high altitude of schizothoracine fishes. Even though it is possible that these new genes may have evolved too fast only in schizothoracine fish lineage to be detected in other fishes, or represent ancestral genes that have lost function in other fishes and accumulated substitutions too fast to be identified as homologs by standard BLAST searches, they are still important and interesting, as fast evolution itself may be an adaptive process. These new genes should be important targets in future studies aiming at elucidating the genetic basis of adaptation to highland of fishes. In addition to de novo genes, gene gain can also be mediated by duplication. However, we could not infer such recent duplication event considering that there is no genome sequence. Therefore, a more thorough understanding of the number and function of genes lost and gained within schizothoracine fishes can only be achieved by increasing taxon sampling and whole genome sequencing.

In addition to loss and gain of genes, adaptive evolution may prefer to occur at the molecular level, expressed by an increased rate of nonsynonymous substitutions to synonymous substitutions (Bakewell et al. 2007). The major adaptations to highland habitat of different endothermic organisms are expansion of gene families, increased evolutionary rate, and positive selection on genes associated with hypoxia response and energy metabolism (Qiu et al. 2012; Ge et al. 2013; Qu et al. 2013). Species living in similar ecological environment can be shaped by convergent evolution to form physiological or morphological similarities (Stern 2013). Just like previous studies in endothermic animals, our evolutionary analyses suggested that the schizothoracine fish can also be characterized by its adaptation to the extreme environment of the Tibetan plateau at the molecular level. First, the schizothoracine fish lineage showed genome-wide accelerated evolution relative to other fish lineages, which is independent of the data set used. Thus, the schizothoracine fishes may have adaptively speeded up their evolutionary rates of genes overall to better adapt to the extreme environment of the Tibetan Plateau, as accelerated evolution is usually driven by positive selection. It is also possible that accelerated evolution could be caused by relaxation of functional constraint, which yet needs to be further confirmed from population genomic analyses in future. Second, only the terminal branch of the schizothoracine fishes had undergone elevated evolutionary rates than the ancestral branch, suggesting that accelerated evolution only occurred in the schizothoracine fish lineage after split from zebrafish. Third, functional GO categories related to hypoxia response and energy metabolism were found to have evolved faster in the schizothoracine fish lineage. Fourth, rapidly evolving and positively selected genes in the schizothoracine fish lineage were also enriched in categories involved in energy metabolism and hypoxia. All in all, these results indicated that the schizothoracine fishes may have experienced adaptive evolution to cope with the extremely inhospitable environment. However, our current evidence only showed accelerated protein sequence evolution in *G. pachycheilus* and whether gene content evolution (gene losses and gains) is also accelerated remains as an interesting question in the next stage.

The most extreme challenge for species living in high-altitude is low oxygen supply (Beall 2007). To identify the potential genes directly involved in hypoxia, we focused on the function of PSGs in the schizothoracine fish lineage and found several interesting candidate genes that may be involved in response to hypoxia. For example, the BYSL gene that encodes an accessory protein for cell adhesion significantly upregulated induced by hypoxia, suggesting an important role in hypoxia response (Fang et al. 2008). The activation of heat shock proteins (HSPs) is critical to adaptation to hypoxia, which is regulated by HSF1. And HSF1 is upregulated directly by HIF-1, suggesting a link of PSGs to hypoxia pathway (Baird et al. 2006). YES1, a member of the Src family of tyrosine protein kinases acting on focal adhesions and contacts (Lynch et al. 2004; Gaudreault et al. 2005), has a relation to HSP27 (Hansen et al. 2001), which can be specifically unregulated by hypoxic signaling through HIF-1 (Whitlock et al. 2005). Genes, ARRDC2 and SSPN are reported to be related to hypoxia and collected in the database of hypoxia-regulated proteins (HypoxiaDB) (Khurana et al. 2013). SEMA4D can promote angiogenesis acting through Plexin-B1 on endothelial cells, which is regulated by HIF-1 (Sun et al. 2009). VWF, an adhesive glycoprotein that expressed exclusively in endothelial cells, increased expression levels in pulmonary hypertension caused by hypoxia (Caramuru et al. 2003; Mojiri et al. 2013). Although there are several candidate genes that are potentially involved in hypoxia showing signature of positive selection, none is shared with previously reported genes in other endothermic animals. This observation suggests that fishes may have employed different genic toolkit to adapt to the extreme environment of the Tibetan Plateau. However, this hypothesis needs to be further confirmed by population genomics in future.

## Supplementary Material

Supplementary tables S1–S9 and figures S1–S7 are available at *Genome Biology and Evolution* online (http://www.gbe.oxfordjournals.org/).

Supplementary Data

## References

[evu279-B1] Altschul SF (1997). Gapped BLAST and PSI-BLAST: a new generation of protein database search programs. Nucleic Acids Res..

[evu279-B2] Axelsson E (2013). The genomic signature of dog domestication reveals adaptation to a starch-rich diet. Nature.

[evu279-B3] Baird NA, Turnbull DW, Johnson EA (2006). Induction of the heat shock pathway during hypoxia requires regulation of heat shock factor by hypoxia-inducible factor-1. J Biol Chem..

[evu279-B4] Bakewell MA, Shi P, Zhang J (2007). More genes underwent positive selection in chimpanzee evolution than in human evolution. Proc Natl Acad Sci U S A..

[evu279-B5] Beall CM (2007). Two routes to functional adaptation: Tibetan and Andean high-altitude natives. Proc Natl Acad Sci U S A. 104(Suppl.

[evu279-B6] Beall CM (2010). Natural selection on EPAS1 (HIF2alpha) associated with low hemoglobin concentration in Tibetan highlanders. Proc Natl Acad Sci U S A..

[evu279-B7] Bickler PE, Buck LT (2007). Hypoxia tolerance in reptiles, amphibians, and fishes: life with variable oxygen availability. Annu Rev Physiol..

[evu279-B8] Bigham A (2010). Identifying signatures of natural selection in Tibetan and Andean populations using dense genome scan data. PLoS Genet..

[evu279-B9] Cai Q (2013). Genome sequence of ground tit *Pseudopodoces humilis* and its adaptation to high altitude. Genome Biol..

[evu279-B10] Cao W, Chen Y, Wu Y, Zhu S (1981). Origin and evolution of schizothoracine fishes in relation to the upheaval of the Xizang Plateau.

[evu279-B11] Caramuru LH, Soares Rde P, Maeda NY, Lopes AA (2003). Hypoxia and altered platelet behavior influence von Willebrand factor multimeric composition in secondary pulmonary hypertension. Clin Appl Thromb Hemost..

[evu279-B12] Castresana J (2000). Selection of conserved blocks from multiple alignments for their use in phylogenetic analysis. Mol Biol Evol..

[evu279-B13] Chen S, Krinsky BH, Long M (2013). New genes as drivers of phenotypic evolution. Nat Rev Genet..

[evu279-B14] Chen Y, Cao W, Yue P (2000). Schizothoracinae.

[evu279-B15] Dennis G (2003). DAVID: Database for Annotation, Visualization, and Integrated Discovery. Genome Biol..

[evu279-B16] Ding Y, Zhou Q, Wang W (2012). Origins of new genes and evolution of their novel functions. Annu Rev Ecol Evol Syst..

[evu279-B17] Durinck S (2005). BioMart and Bioconductor: a powerful link between biological databases and microarray data analysis. Bioinformatics.

[evu279-B18] Ellegren H (2012). The genomic landscape of species divergence in *Ficedula flycatchers*. Nature.

[evu279-B19] Fang D (2008). Expression of bystin in reactive astrocytes induced by ischemia/reperfusion and chemical hypoxia in vitro. Biochim Biophys Acta..

[evu279-B20] Ferreira PG (2013). Transcriptome analyses of primitively eusocial wasps reveal novel insights into the evolution of sociality and the origin of alternative phenotypes. Genome Biol..

[evu279-B21] Flicek P (2013). Ensembl 2013. Nucleic Acids Res..

[evu279-B22] Gaudreault E, Thompson C, Stankova J, Rola-Pleszczynski M (2005). Involvement of BLT1 endocytosis and Yes kinase activation in leukotriene B4-induced neutrophil degranulation. J Immunol..

[evu279-B23] Ge RL (2013). Draft genome sequence of the Tibetan antelope. Nat Commun..

[evu279-B24] Goodman M (2009). Phylogenomic analyses reveal convergent patterns of adaptive evolution in elephant and human ancestries. Proc Natl Acad Sci U S A..

[evu279-B25] Gotz S (2008). High-throughput functional annotation and data mining with the Blast2GO suite. Nucleic Acids Res..

[evu279-B26] Gou X (2014). Whole-genome sequencing of six dog breeds from continuous altitudes reveals adaptation to high-altitude hypoxia. Genome Res..

[evu279-B27] Grabherr MG (2011). Full-length transcriptome assembly from RNA-Seq data without a reference genome. Nat Biotechnol..

[evu279-B28] Guo B, Chain FJ, Bornberg-Bauer E, Leder EH, Merila J (2013). Genomic divergence between nine- and three-spined sticklebacks. BMC Genomics.

[evu279-B29] Guo B, Zou M, Wagner A (2012). Pervasive indels and their evolutionary dynamics after the fish-specific genome duplication. Mol Biol Evol..

[evu279-B30] Hahn MW, Demuth JP, Han SG (2007). Accelerated rate of gene gain and loss in primates. Genetics.

[evu279-B31] Hansen RK, Parra I, Hilsenbeck SG, Himelstein B, Fuqua SAW (2001). Hsp27-induced MMP-9 expression is influenced by the Src tyrosine protein kinase Yes. Biochem Biophys Res Commun..

[evu279-B32] He DK, Chen YF (2006). Biogeography and molecular phylogeny of the genus *Schizothorax* (Teleostei : Cyprinidae) in China inferred from cytochrome b sequences. J Biogeogr..

[evu279-B33] He DK, Chen YF, Chen YY, Chen ZM (2004). Molecular phylogeny of the specialized schizothoracine fishes (Teleostei : Cyprinidae), with their implications for the uplift of the Qinghai-Tibetan Plateau. Chin Sci Bull..

[evu279-B34] Huang da W, Sherman BT, Lempicki RA (2009). Systematic and integrative analysis of large gene lists using DAVID bioinformatics resources. Nat Protoc..

[evu279-B35] Jones FC (2012). The genomic basis of adaptive evolution in threespine sticklebacks. Nature.

[evu279-B36] Kent WJ (2002). BLAT—the BLAST-like alignment tool. Genome Res..

[evu279-B37] Khurana P, Sugadev R, Jain J, Singh SB (2013). HypoxiaDB: a database of hypoxia-regulated proteins. Database (Oxford).

[evu279-B38] Li W, Godzik A (2006). Cd-hit: a fast program for clustering and comparing large sets of protein or nucleotide sequences. Bioinformatics.

[evu279-B39] Li Y (2014). Population variation revealed high-altitude adaptation of tibetan mastiffs. Mol Biol Evol..

[evu279-B40] Loytynoja A, Goldman N (2005). An algorithm for progressive multiple alignment of sequences with insertions. Proc Natl Acad Sci U S A..

[evu279-B41] Lynch G (2004). The tyrosine kinase Yes regulates actin structure and secretion during pancreatic acinar cell damage in rats. Pflugers Arch..

[evu279-B42] Mojiri A (2013). Hypoxia results in upregulation and de novo activation of von Willebrand factor expression in lung endothelial cells. Arterioscler Thromb Vasc Biol..

[evu279-B43] Near TJ (2012). Resolution of ray-finned fish phylogeny and timing of diversification. Proc Natl Acad Sci U S A..

[evu279-B44] Peng Y (2011). Genetic variations in Tibetan populations and high-altitude adaptation at the Himalayas. Mol Biol Evol..

[evu279-B45] Poelstra JW (2014). The genomic landscape underlying phenotypic integrity in the face of gene flow in crows. Science.

[evu279-B46] Qiu Q (2012). The yak genome and adaptation to life at high altitude. Nat Genet..

[evu279-B47] Qu Y (2013). Ground tit genome reveals avian adaptation to living at high altitudes in the Tibetan plateau. Nat Commun..

[evu279-B48] Rice P, Longden I, Bleasby A (2000). EMBOSS: the European Molecular Biology Open Software Suite. Trends Genet..

[evu279-B49] Schunter C, Vollmer SV, Macpherson E, Pascual M (2014). Transcriptome analyses and differential gene expression in a non-model fish species with alternative mating tactics. BMC Genomics.

[evu279-B50] Simonson TS (2010). Genetic evidence for high-altitude adaptation in Tibet. Science.

[evu279-B51] Smith NG, Eyre-Walker A (2002). Adaptive protein evolution in *Drosophila*. Nature.

[evu279-B52] Soria-Carrasco V (2014). Stick insect genomes reveal natural selection’s role in parallel speciation. Science.

[evu279-B53] Steinke D, Salzburger W, Meyer A (2006). Novel relationships among ten fish model species revealed based on a phylogenomic analysis using ESTs. J Mol Evol..

[evu279-B54] Stern DL (2013). The genetic causes of convergent evolution. Nat Rev Genet..

[evu279-B55] Storey JD, Tibshirani R (2003). Statistical significance for genomewide studies. Proc Natl Acad Sci U S A..

[evu279-B56] Su J (2014). Genetic structure and demographic history of the endangered and endemic schizothoracine fish *Gymnodiptychus pachycheilus* in Qinghai-Tibetan Plateau. Zool Sci..

[evu279-B57] Sun Q, Zhou H, Binmadi NO, Basile JR (2009). Hypoxia-inducible factor-1-mediated regulation of semaphorin 4D affects tumor growth and vascularity. J Biol Chem..

[evu279-B58] Thompson LG (2000). A high-resolution millennial record of the south asian monsoon from himalayan ice cores. Science.

[evu279-B59] Wang L (2013). CPAT: Coding-Potential Assessment Tool using an alignment-free logistic regression model. Nucleic Acids Res..

[evu279-B60] Wang S, Xie Y (2004). China species red list.

[evu279-B61] Whitlock NA, Agarwal N, Ma JX, Crosson CE (2005). Hsp27 upregulation by HIF-1 signaling offers protection against retinal ischemia in rats. Invest Ophthalmol. Vis Sci..

[evu279-B62] Wu B, Deng Y, Wu J, Yan C, Song Z (2014). Complete mitochondrial genome of *Gymnodiptychus pachycheilus* (Teleostei: Cypriniformes: Cyprinidae). Mitochondrial DNA.

[evu279-B63] Wu Y, Wu C (1991). The fishes of the Qinghai—Xizang plateau.

[evu279-B64] Xing J (2013). Genomic analysis of natural selection and phenotypic variation in high-altitude mongolians. PLoS Genet..

[evu279-B65] Xu S (2011). A genome-wide search for signals of high-altitude adaptation in Tibetans. Mol Biol Evol..

[evu279-B66] Yang Z (2007). PAML 4: phylogenetic analysis by maximum likelihood. Mol Biol Evol..

[evu279-B67] Yi X (2010). Sequencing of 50 human exomes reveals adaptation to high altitude. Science.

[evu279-B68] Zhang J, Nielsen R, Yang Z (2005). Evaluation of an improved branch-site likelihood method for detecting positive selection at the molecular level. Mol Biol Evol..

[evu279-B69] Zhang W (2014). Hypoxia adaptations in the grey wolf (*Canis lupus chanco*) from Qinghai-Tibet Plateau. PLoS Genet..

[evu279-B70] Zhao S (2013). Whole-genome sequencing of giant pandas provides insights into demographic history and local adaptation. Nat Genet..

