# Supplementary material for: Comprehensive Transcriptome Analysis Reveals Accelerated Genic Evolution in a Tibet Fish, Gymnodiptychus pachycheilus
Source: Genome Biol Evol. 2014 Dec 26;7(1):251–61. doi: 10.1093/gbe/evu279 (PMC4316632; doi:10.1093/gbe/evu279)
Supplement: Supplementary Data [file supp_7_1_251__index.html]

Comprehensive transcriptome analysis reveals accelerated genic evolution in a Tibet fish, Gymnodiptychus pachycheilus — Comprehensive Transcriptome Analysis Reveals Accelerated Genic Evolution in a Tibet Fish, Gymnodiptychus pachycheilus — Supplementary Data 

# Comprehensive Transcriptome Analysis Reveals Accelerated Genic Evolution in a Tibet Fish, *Gymnodiptychus pachycheilus*

## Supplementary Data

files

**Files in this Data Supplement:**

- Supplementary Data - docx file
- Supplementary Data - xlsx file
