# Supplementary material for: Comprehensive Transcriptome Analysis Reveals Accelerated Genic Evolution in a Tibet Fish, Gymnodiptychus pachycheilus
Source: Genome Biol Evol. 2014 Dec 26;7(1):251–61. doi: 10.1093/gbe/evu279 (PMC4316632; doi:10.1093/gbe/evu279)

**Supplementary Figures**

**Fig. S1.** Length distribution of unigenes.

**Fig. S2.** The relationship between length of unigenes and number of reads.


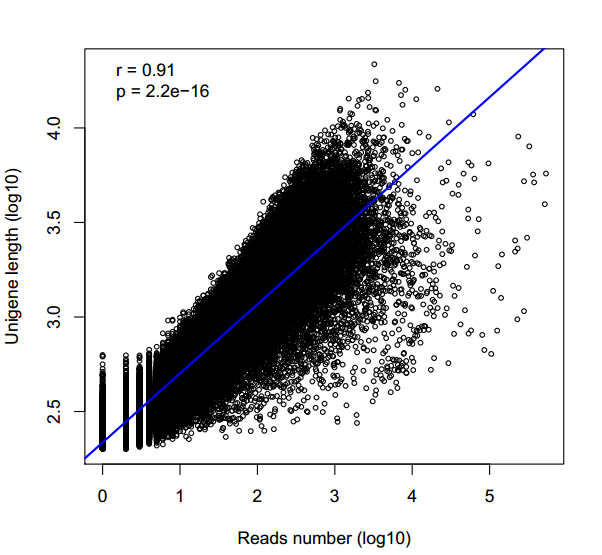


**Fig. S3.** The results of RT-PCR. The products of RT-PCR were sorted by their length.

**
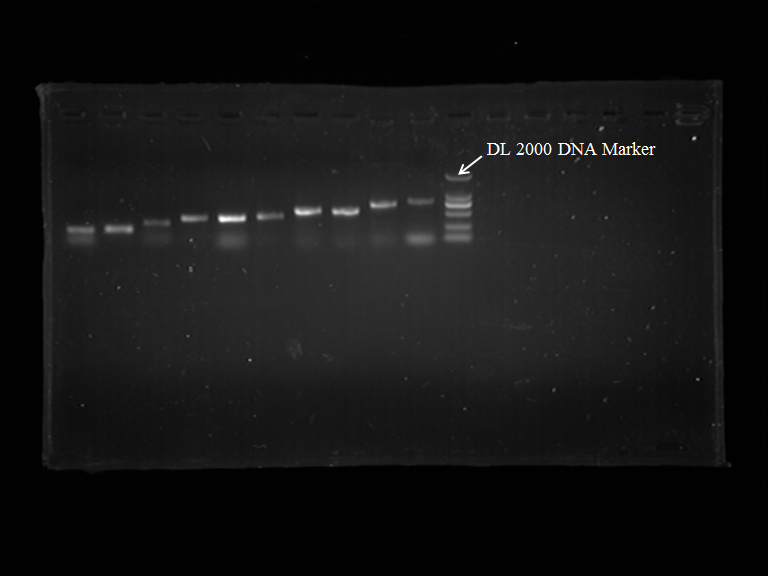
**

**Fig. S4.** Functional annotation of assembled unigenes in *G. pachycheilus* based on Gene Ontology (GO) categorization.


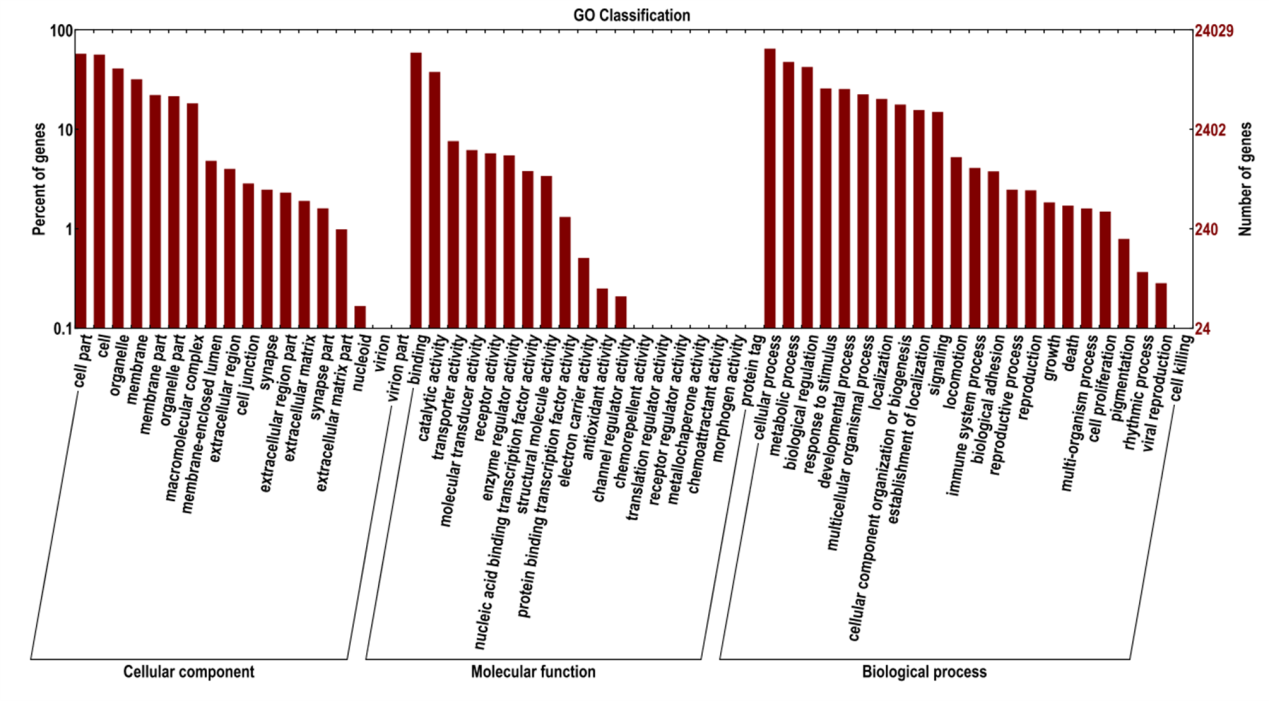


**Fig. S5.** COG annotations of putative proteins.


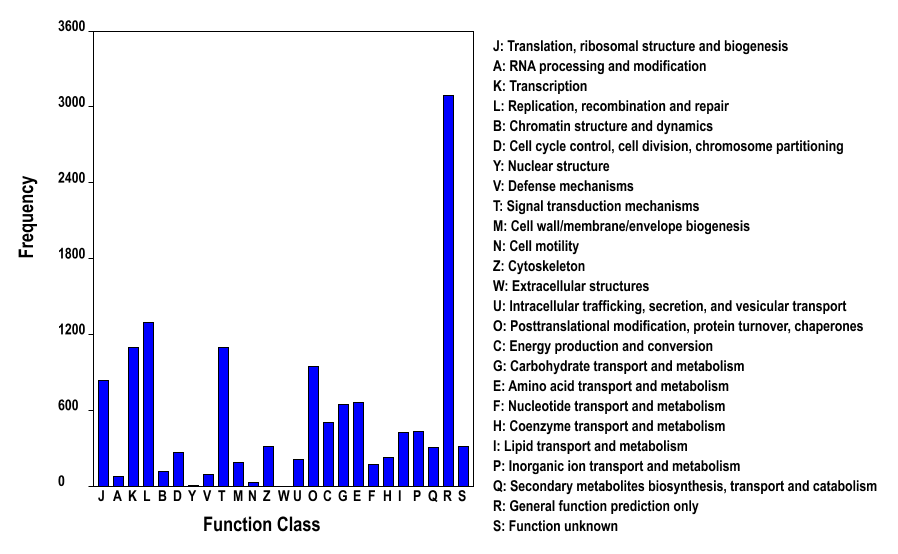


**Fig. S6.** Statistics of annotation of unigenes to the GenBank non-redundant (NR) database.


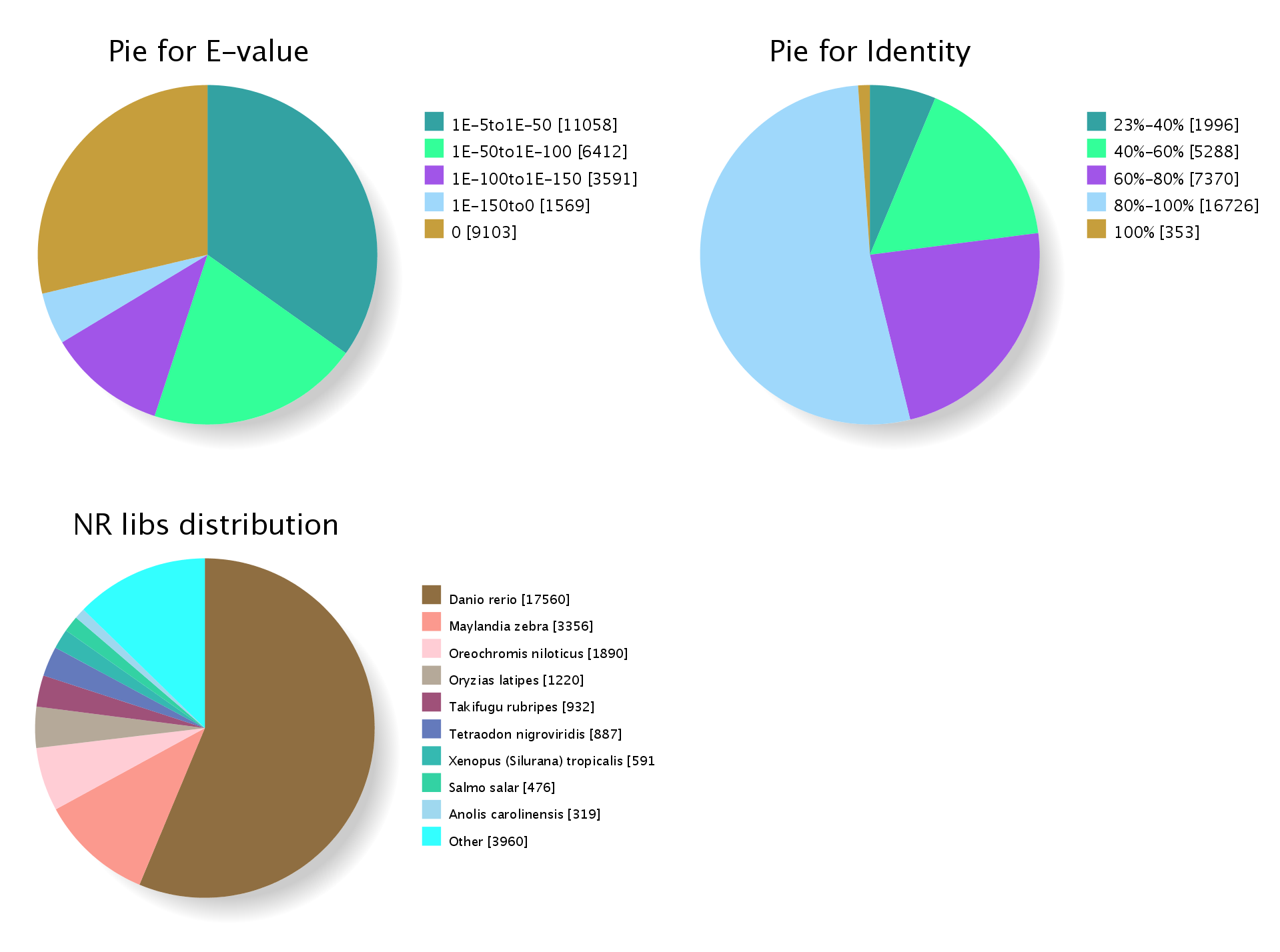


**Fig. S7.** The length distribution of orthologs before (A, B) and after (C, D) trimming. (A, C) The length distribution of all orthologs; (B, D) The length distribution of orthologs with length from 1 to 5,000.


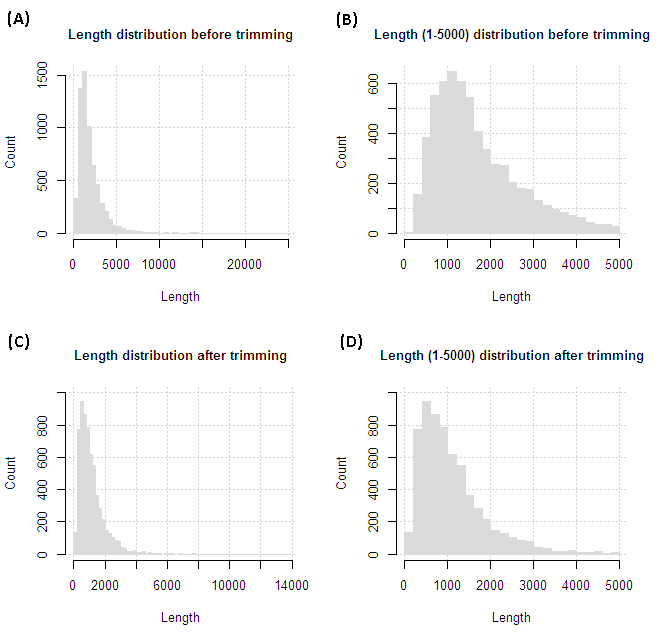

Supplement: Supplementary Data [file supp_evu279_Supplementary_Figures.docx]
